# Fine-scale genetic analysis of the exploited Nile monitor (*Varanus niloticus*) in Sahelian Africa

**DOI:** 10.1186/s12863-015-0188-x

**Published:** 2015-03-28

**Authors:** Stephanie A Dowell, Vivian de Buffrénil, Sergios-Orestis Kolokotronis, Evon R Hekkala

**Affiliations:** Department of Biological Sciences, Fordham University, 441 East Fordham Road, 10458 Bronx, NY USA; Laboratoire d’Anatomie comparée, Muséum National d’Histoire Naturelle, 55 rue Buffon, 75005 Paris, France

**Keywords:** Varanid, Population genetics, Genetic structure, Genetic diversity, Effective population size, Inner Niger delta, Exploitation, Population decline

## Abstract

**Background:**

Overexploitation of wildlife populations results in direct consequences, such as extinction and local extirpation, as well as indirect effects including genetic diversity loss and changes in genetic structure. A clear understanding of the underlying genetic patterns of harvested species is necessary for sustainable management. The Nile monitor (*Varanus niloticus*) is a commercially valuable species in the international leather industry, with the highest levels of exploitation concentrated throughout Sahelian Africa. In this study, we examined the fine-scale genetic patterns of *V. niloticus* populations in the Sahel, with the expectation that the genetic structure would correspond to riverine drainage basins. The analyses were based on genotypes at 11 microsatellite loci for 318 individuals, spanning three separate watersheds throughout the Sahel.

**Results:**

Our analyses identified four genetic clusters throughout the region, one of which (the westernmost population) exhibited very high levels of genetic differentiation (*F*_*ST*_ = 0.47). Contrary to our expectation, the largest genetic break occurred within a single watershed, the Niger basin, rather than between watersheds. However, other localities displayed evidence of reduced gene flow between watershed boundaries. Across methods, the westernmost population exhibited lower estimates of *N*_*e*_ as well as lower levels of genetic diversity compared to the other inferred populations. While we did not detect evidence for recent population bottlenecks, our analyses indicated historic population declines around 1,000–1,800 years ago.

**Conclusion:**

We found that the underlying genetic structure of *Varanus niloticus* across Sahelian Africa reflects historic as well as present-day patterns of riverine drainages. The high degree of differentiation found for the westernmost population indicates the presence of a separate lineage, and should be taken into consideration when setting harvest limits. The historic population decline for two of the populations corresponds to a drastic expansion of an ancient human civilization in the region, suggesting that human exploitation of *V. niloticus* has a longer history than previously thought.

**Electronic supplementary material:**

The online version of this article (doi:10.1186/s12863-015-0188-x) contains supplementary material, which is available to authorized users.

## Background

Overexploitation has been identified as the main source of decline in many threatened species worldwide [1]. Direct effects of overharvesting are well-supported, including population declines, extirpation, and even extinction [2]. However, indirect consequences of exploitation, such as changes in genetic diversity and population structure, must also be considered in order for species to be sustainably harvested [3]. Overharvesting can alter genetic subdivisions and gene flow patterns, either leading to population isolation and genetic drift, or to increased gene flow resulting in genetic swamping and the loss of local adaptations [3]. By targeting a specific age class or sex, harvesting can reduce the effective population size (*N*_*e*_), and thus the genetic diversity, without affecting the overall population size [4]. Even non-specific hunting can cause significant genetic and morphological changes in a population [5].

The Nile monitor (*Varanus niloticus*) is the second most heavily traded varanid species in both the leather and the pet industries [6,7]. Reaching lengths of over two meters, *V. niloticus* is also commonly used as a source of food in some regions [8]. The distribution of this species encompasses most of sub-Saharan Africa and additionally extends northward along the Nile River into Egypt [9]. Due to their semi-aquatic nature, the only consistent habitat requirement throughout their range is the presence of permanent bodies of water [9,10]. The diet of *V. niloticus* is entirely carnivorous, preying upon a large variety of organisms from snails and insects to small mammals [9].

While the distribution of *Varanus niloticus* is widespread across Africa, the major skin exporters are concentrated in the Sahelian region, specifically Mali, Cameroon, Chad, Nigeria, and Sudan [6,11,12]. Approximately 500,000 *V. niloticus* skins legally enter international trade annually to be used for shoes, wallets, belts, handbags, and watchstraps [6,8,11,12]. Accounting for damaged skins and undocumented trade, harvest estimates in the Sahelian region alone have reached two million in peak years [8]. The pet trade adds additional pressure to *V. niloticus* populations. Between 1975 and 2005, live exports of *V. niloticus* constituted 22.9% of the global trade in varanids, totaling 309,759 individuals [7].

Although *Varanus niloticus* displays high fecundity (average of 20 eggs per clutch) and relatively early sexual maturation (around two years of age), intense exploitation could be affecting their populations [9,13]. Highly exploited populations in eastern Mali were shown to exhibit earlier sexual maturation, higher reproductive output, and shorter longevity than populations near Lake Chad with lower harvest rates [13,14]. A study by de Buffrénil and Hémery [14] additionally reported that *V. niloticus* individuals collected near Lake Chad after years of intense harvesting were significantly smaller than those collected in a similar location years earlier when harvesting was minimal. Furthermore, the combined effects of harvesting *V. niloticus* for food, leather, and the pet trade are likely leading to local extirpation of subpopulations [12]. However, unless the genetic subdivisions within this species are identified, recognizing or preventing the loss of genetically unique populations will not be possible.

While *Varanus niloticus* is protected under the Convention on International Trade in Endangered Species of Wild Fauna and Flora (CITES, Appendix II), little is known about the population size and genetic structure of this species, which hinders effective management decisions [11]. This study examines the genetic structure, effective population size, and dispersal patterns of *V. niloticus* populations throughout Sahelian Africa, where harvesting is heavily concentrated. Due to the semi-aquatic behavior and habitat requirements of this species [9], we expected the genetic partitioning within *V. niloticus* to correspond to river drainage basins throughout the region.

## Methods

### Ethics statement

All tissue samples used in this study were collected and transported in compliance with the Convention on International Trade in Endangered Species of Fauna and Flora.

### Sample collection and DNA extraction

We obtained 434 *Varanus niloticus* tissue samples collected from 18 separate localities throughout Sahel (Table 1). Collection dates ranged from 1991 through 2009 and were largely dependent on local hunters (see de Buffrénil and Hémery [14] for a more detailed description of collection methods). All Chad localities except Fodiawalli near Lake Chad had been subjected to intense harvest rates for at least five years before sample collection [14]. Additionally, *V. niloticus* populations in Mali have been under constant, high levels of exploitation for a prolonged period of time [14].

**Table 1:** **Collection locality information and number of samples (** ***N*** **) for** ***Varanus niloticus*** **throughout Sahelian Africa**

**Sample Locality**	**Country**	**Drainage Basin**	**Year Collected**	***N*** **(Collected)**	***N*** **(Included in Analyses)**
1. Flabougou	Mali	Senegal	2008	13	13
2. Niono	Mali	Niger	2008	18	18
3. Mopti	Mali	Niger	1991/1996	51	21 (1996 Only)
4. Niamey	Niger	Niger	2009	3	3
5. Lake Lere	Chad	Niger	1997	15	12
6. Diffa	Niger	Lake Chad	2009	15	15
7. Lake Chad: Djaloua	Chad	Lake Chad	1997	57	57
8. Lake Chad: Garaerem	Chad	Lake Chad	1997	61	43
9. Lake Chad: Fodio	Chad	Lake Chad	1997	15	15
10. Lake Chad: Kambé	Chad	Lake Chad	1997	15	15
11. Lake Chad: Moussarom	Chad	Lake Chad	1996	15	15
12. Lake Chad: Fodiawalli	Chad	Lake Chad	1991	30	29
13. Lake Chad: Koutkou	Chad	Lake Chad	2003	15	15
14. Abba Liman	Chad	Lake Chad	2002	5	5
15. Lake Fitri	Chad	Lake Chad	1997	33	33
16. Am N’Guitey	Chad	Lake Chad	2002	9	9
Lake Chad: Banangore (Excluded)	Chad	Lake Chad	1990	27	0
North of Garoua (Excluded)	Cameroon	Niger	1992	37	0
Total				434	318

**Figure 1:**
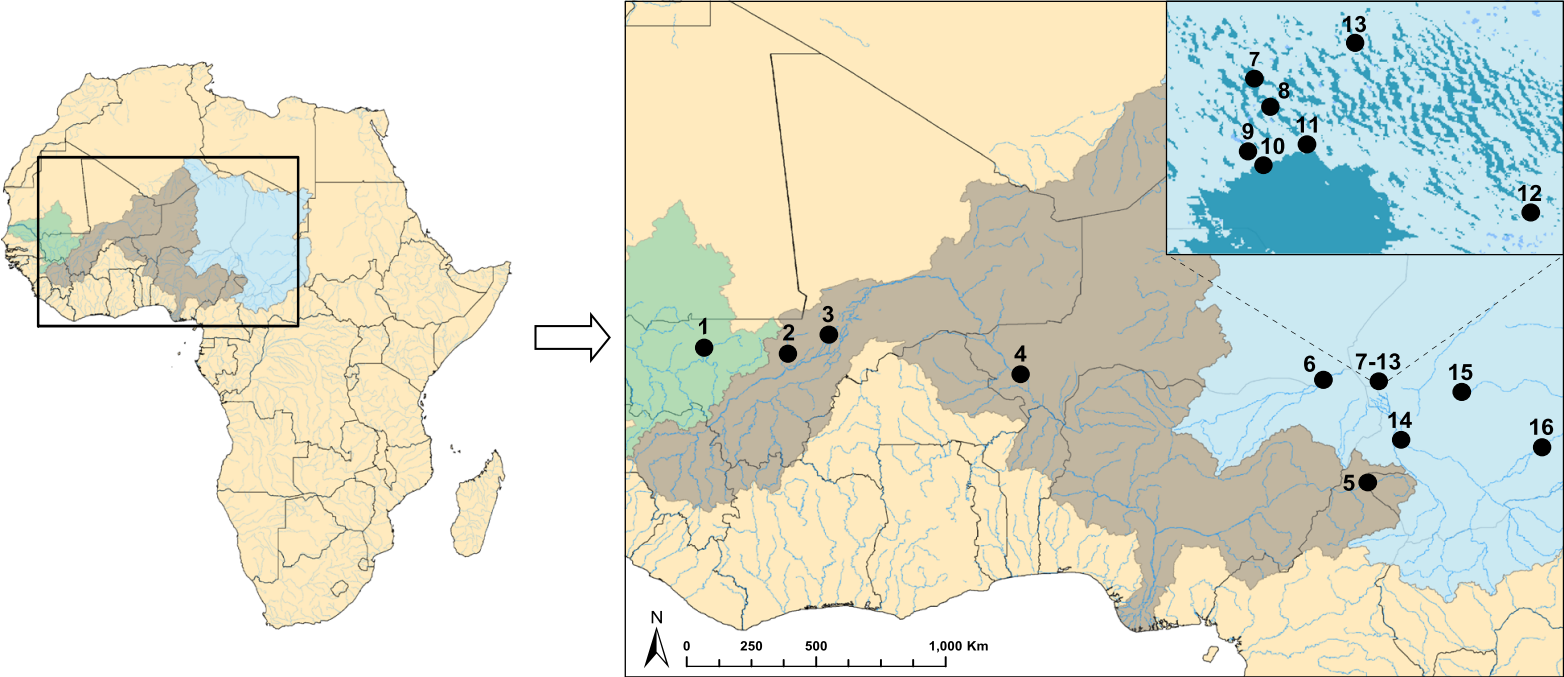
**Map of Africa showing the 16** ***Varanus niloticus*** **sample localities analyzed in this study.** The site numbers correspond to those on Table 1. The Senegal, Niger, and Lake Chad drainage basins are highlighted in green, gray, and blue, respectively.

Sample sites are located in three separate drainage basins: the Senegal, Niger, and Lake Chad basins. Additionally, in the Lake Chad basin, sites 7–13 are located in the vicinity of Lake Chad itself, while four sites (6, 14–16) are located along tributaries (Figure 1).

A 1 cm^2^ section of tissue was removed from each sample of dried muscle/ bone, sterilizing instruments between sampling with DNA*Away*^TM^ to prevent cross contamination. Genomic DNA extraction was performed using the Qiagen DNeasy® Blood and Tissue Kit, following manufacturer’s protocol for tissue.

### Microsatellite screening and genotyping

Using a subset of *Varanus niloticus* DNA extracts, we screened 29 microsatellite loci identified in congeners *V. komodoensis,* abbreviated “K” [15,16], *V. salvator*, abbreviated “VARSA” [17], and *V. acanthurus*, abbreviated “VA” [18]. Of these loci, 11 amplified consistently and were polymorphic in *V. niloticus*: *K7*, *K10*, *K11*, *K15*, *K22*, *K23*, *VARSA10*, *VARSA07*, *VA17*, *VA38*, and *VA74*.

PCR reactions contained 10.0 ng DNA template, 0.4 μM fluorescently-labeled forward primer, 0.4 mM reverse primer, and 1X AmpliTaq Gold® 360 Master Mix in a final reaction volume of 13.75 μL. PCR amplifications were performed in Techne TC-5000 or Applied Biosystems 2720 thermocycler and conditions varied by locus (Additional file 1: Table S1). PCR products were visualized on a 1.5% agarose gel to confirm successful amplification. Fragment analysis was carried out using an ABI 3100 Genetic Analyzer with GeneScan™ 500 LIZ® size standard and allele sizes were called using GeneMarker®.

### Microsatellite analysis

The microsatellite data were examined for null alleles and mis-scoring using MICRO-CHECKER [19]. Hardy-Weinberg equilibrium (HWE) and linkage disequilibrium were assessed with the web-based program GENEPOP v.4.2 [20]. We calculated expected heterozygosity (*H*_*e*_), observed heterozygosity (*H*_*o*_), and number of alleles (*N*_*a*_) with GenAlEx v.6.5 [21] and allelic richness (*A*_*R*_) and private allelic richness (*PA*_*R*_) were calculated with HP-Rare 1.1 [22] which uses a rarefaction method to correct for large differences in sample size.

### Genetic structure

We used multiple approaches to examine the genetic partitioning among *Varanus niloticus* populations in Sahelian Africa. We measured genetic differentiation (*F*_*ST*_) among sample localities in ARLEQUIN 3.5.1.3 [23] using 10,100 permutations to test for significance. Principal Coordinate Analysis (PCoA) based on the calculated pairwise *F*_*ST*_ values was conducted in GENALEX v.6.5 [21] and plotted in R v.3.1 [24,25] to visualize the relationships among localities.

Bayesian clustering analyses were performed using STRUCTURE v.2.3.3 [26] and BAPS v.6.0 [27]. STRUCTURE groups individuals into randomly mating populations by maximizing Hardy-Weinberg and linkage equilibrium [26]. We used a correlated allele frequency model with admixture and did not incorporate sampling locality. Ten runs were carried out for each *K* value, from 1 to 10, with an initial burn-in of 10^6^ iterations and an additional 10^7^ iterations after the burn-in. The optimal number of clusters (*K*) was estimated by examining the Ln P(X|*K*) [28] as well as the Δ*K* [29] with the web-based program STRUCTURE HARVESTER [30]. For each *K* value analyzed, the results were averaged across runs using CLUMPP v.1.1.2 [31]. For comparison, we also performed a spatial clustering of individuals with BAPS v.6.0 [27], followed by an admixture analysis with 10^7^ iterations, 100 reference individuals, and 1,000 iterations of reference individuals [32]. BAPS directly infers the number of populations from the dataset without the need for examining multiple *K* values and additional ad-hoc analyses. Results from both analyses were visualized with DISTRUCT v.1.1 [33].

A hierarchical analysis of molecular variance (AMOVA) was carried out in ARLEQUIN v.3.5.1.3 [23] to determine the significance of the genetic partitioning.

### Effective population size and demographic changes

For the following set of analyses, we grouped samples into their inferred populations based on the results from the clustering methods. We implemented MSVAR v.1.3 [34] for our dataset, which estimates effective population size (*N*_*e*_) as well as assesses historical demographic changes by applying a Bayesian coalescent-based technique. Ten separate runs were conducted for each of the inferred populations using different starting points and 2.0x10^4^ thinned updates with a thinning interval of 1.0x10^4^. Following de Buffrénil and Rimblot-Baly [13], the generation time was set to 2 years. The initial 10% of the resulting data points were discarded to avoid starting configuration bias. The remaining data points were concatenated and then plotted in R v.3.1 [24,25] to interpret the results. We also compared the estimates of current *N*_*e*_ with NeESTIMATOR v.2 [35] using the linkage disequilibrium method for random mating [36].

To test for evidence of recent population declines, we used BOTTLENECK v.1.2.02 [37] which tests for deviations from expected heterozygosity under the infinite allele model (IAM), stepwise mutation model (SMM), and two-phase model (TPM) with 70% SMM. The statistical analyses implemented by the program (sign test, standardized differences test, and Wilcoxon sign rank test) were used to test for significant excess in heterozygosity.

### Gene flow and dispersal

To assess the degree of gene flow among the inferred populations, we used BAYESASS v.1.3 [38] with 10^7^ iterations, a burn-in of 10^6^ and sampling every 2,000 iterations.

Lastly, we examined whether differences in gene flow among localities were correlated with river distance and environmental distance. Pairwise river distances between localities were measured in ArcGIS 10.1 [39]. We performed a Mantel test using pairwise *F*_*ST*_/(1-*F*_*ST*_) calculations, following Rousset [40], across all 16 localities using IBDWS v.3.23 [41].To assess the correlation between genetic distance and environmental heterogeneity, we performed a Partial Mantel test with IBDWS and controlled for the influence of river distance. Environmental variables included BIOCLIM layers for annual precipitation, temperature seasonality, and elevation (www.worldclim.org/bioclim), and pairwise environmental distances were calculated as the pixel value difference between each locality.

## Results

### Amplification success

Of the 434 tissue samples originally obtained, 318 individuals from 16 localities successfully amplified at seven or more loci and were included in further analyses. Examination of the data with MICRO-CHECKER [19] indicated no evidence of null alleles. Two loci (*K23* and *VARSA10*) were found to significantly depart from Hardy-Weinberg equilibrium (Additional file 2: Table S2); however, because this result was not consistent across sampling locations, both loci were retained in further analyses. These two loci also exhibited evidence of linkage disequilibrium at one sample locality (site 7), but because no other sample sites showed evidence of non-random associations, the loci were not removed.

### Genetic structure

**Figure 2:**
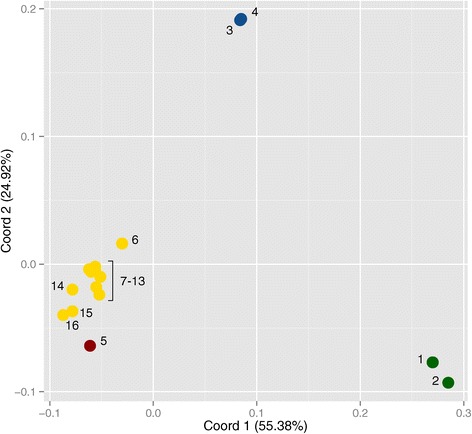
**Principal coordinate analysis based on pairwise** ***F***_***ST***_ **values across all** ***Varanus niloticus*** **sample localities.** Points are labeled with locality numbers and are consistent with Table 1 and Figure 1. The colors represent inferred genetic clusters: green = western, blue = central, red = Lake Lere, and yellow = Lake Chad watershed.

A high degree of genetic partitioning was uncovered among *Varanus niloticus* populations throughout the Sahel. Pairwise *F*_*ST*_ values showed a wide range, with values near zero for geographically close localities near Lake Chad, and reaching 0.58 (site 2 vs. site 5) for western sites (see Table 2). The genetic relationships among sampling localities were more clearly visualized in the principal coordinate analysis (PCoA) in Figure 2. The first two principal coordinates accounted for a total of 80.3% of the variation. The westernmost localities (sites 1 and 2) showed clear separation from the other sites, followed by the more central localities (sites 3 and 4), which grouped together. Interestingly, the eastern sampling locations exhibited genetic relationships consistent with their geographic orientation. Sites 7–13, near Lake Chad, formed a tight cluster, while those along tributaries within the same watershed (sites 6, 14–16) were more diffuse. Lake Lere (site 5), located in the Niger watershed, was not recovered within the Lake Chad cluster.

**Table 2:** **Pairwise** ***F***_***ST***_ **values among** ***Varanus niloticus*** **sample localities**

	**1**	**2**	**3**	**4**	**5**	**6**	**7**	**8**	**9**	**10**	**11**	**12**	**13**	**14**	**15**
**2**	**0.08226**														
**3**	**0.44593**	**0.48545**													
**4**	**0.43163**	**0.49889**	0.00843												
**5**	**0.53828**	**0.58217**	**0.41298**	**0.40193**											
**6**	**0.47528**	**0.50742**	**0.25375**	**0.24334**	**0.19394**										
**7**	**0.44308**	**0.47909**	**0.22774**	**0.23497**	**0.09626**	**0.06499**									
**8**	**0.44818**	**0.48291**	**0.26298**	**0.26256**	**0.09443**	**0.08208**	0.00220								
**9**	**0.44678**	**0.49629**	**0.26165**	**0.24932**	**0.10293**	**0.05896**	0.00094	0.00349							
**10**	**0.45867**	**0.51136**	**0.23647**	**0.24207**	**0.13610**	**0.10636**	0.00490	**0.01652**	0.00555						
**11**	**0.46003**	**0.50963**	**0.24136**	**0.22645**	**0.09334**	**0.07980**	−0.00565	0.00144	0.01671	0.00947					
**12**	**0.46124**	**0.50146**	**0.22482**	**0.23775**	**0.13068**	**0.06445**	0.00477	**0.01957**	0.01578	0.00832	0.00215				
**13**	**0.47271**	**0.52061**	**0.24416**	**0.23657**	**0.10847**	**0.07891**	0.00213	**0.01561**	0.01454	**0.02527**	−0.00198	0.01188			
**14**	**0.49191**	**0.55248**	**0.28106**	**0.27688**	**0.12672**	**0.07898**	0.00781	**0.02977**	0.00988	0.01041	−0.00242	0.00179	−0.00992		
**15**	**0.53076**	**0.56784**	**0.32690**	**0.35277**	**0.18137**	**0.14055**	**0.04055**	**0.05436**	**0.04425**	**0.03452**	**0.05285**	**0.03539**	**0.05507**	0.01257	
**16**	**0.51510**	**0.56095**	**0.31735**	**0.32533**	**0.11112**	**0.10315**	0.00841	0.01564	0.01293	0.01206	−0.00488	0.00591	0.00976	−0.02638	0.01559

Numbering is consistent with localities in Table 1. Bold values indicate significant *P*-values < 0.05, calculated with ARLEQUIN using 10,100 permutations.

The clustering analyses showed a hierarchical pattern of structuring across *Varanus niloticus* sample localities throughout Sahelian Africa. Following the method of Evanno et al. [29], the largest Δ*K* occurred at *K* = 2, followed by a secondary peak at *K* = 4 (Additional file 3: Figure S1). Additionally, the plot of Ln P(X|*K*) did not begin to plateau until *K* = 4. We interpreted these results to mean that while the largest genetic break occurred at *K* = 2, the optimal number of genetic clusters was four. This decision was bolstered by the BAPS result which also produced four clusters.

**Figure 3:**
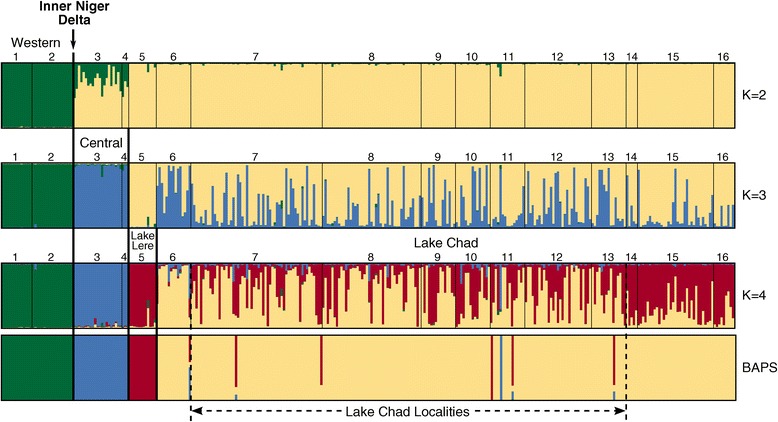
**Genetic structure among** ***Varanus niloticus*** **localities based on Bayesian cluster analyses.** STRUCTURE results, showing *K* = 2–4, and BAPS results are shown. Locality numbers correspond to those in Figure 1 and inferred populations are indicated.

**Figure 4:**
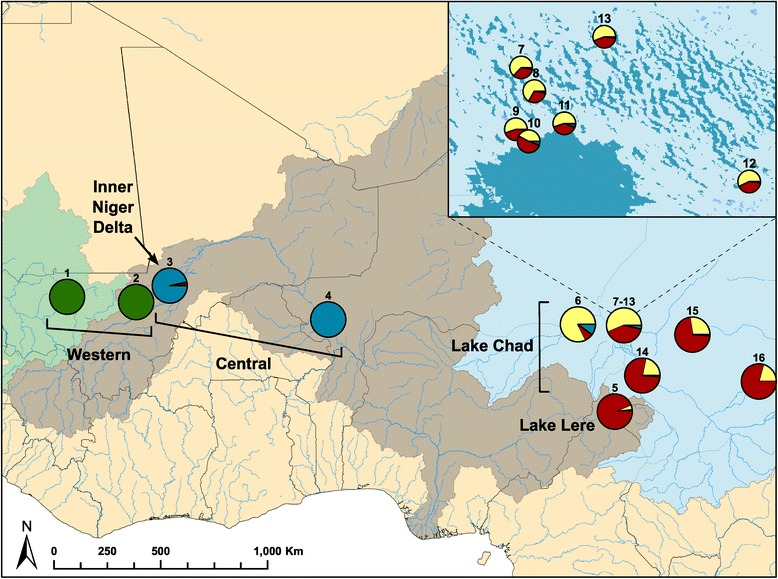
**Map showing the proportions of ancestry for all sample localities to each of the inferred genetic clusters.** Proportions are based on the STRUCTURE results for *K* = 4, averaged across 10 runs. Locality numbers correspond to those in Table 1 and the groupings refer to the inferred populations based on clustering methods.

The largest degree of genetic partitioning, occurring at *K* = 2, separated the two westernmost localities from the remaining sites (Figures 3 and 4). Contrary to our original hypothesis, this genetic break occurs within a single drainage basin, the Niger basin, and separates localities to the west and east of the Inner Niger Delta, a large network of interconnecting streams and rivers. The next genetic break, at *K* = 3, separates localities in the western portion of the Niger basin (sites 3 and 4 in the Niger River) from the eastern portion (site 5 along the Benue River). Lastly, at *K* = 4, genetic differentiation between the Niger and Lake Chad drainage basins becomes apparent and is more clearly demarcated in the BAPS analysis. Showing patterns consistent with the PCoA, the STRUCTURE analysis produced additional sub-structuring in the Lake Chad drainage basin.

Hierarchal analysis of molecular variance (AMOVA) confirmed that the largest amount of genetic variation can be explained by grouping localities into west versus east of the Inner Niger Delta. Groups based on genetic cluster assignment as well as drainage basins also explained a significant amount of the total genetic variation (Table 3).

**Table 3:** **Hierarchical AMOVA results for** ***Varanus niloticus*** **populations across Sahelian Africa**

**Source of Variation**	**Sum of Squares**	**Variance Components**	**Percentage of Variation**	***F***_***ST***_	***P*** **-value**
Between West vs. East Inner Delta	200.269	1.71099	42.40	0.47147	0.0000
Among Populations within Groups	133.853	0.19169	4.75		
Within Populations	1322.457	2.13299	52.85		
Total	1656.579	4.03567			
Among Genetic Clusters	277.735	1.15560	34.46	0.36389	0.0000
Among Populations within Clusters	56.387	0.06457	1.93		
Within Populations	1322.457	2.13299	63.61		
Total	1656.579	3.35316			
Among Watersheds	169.032	0.66580	21.70	0.30477	0.0000
Among Populations within Watersheds	165.090	0.26923	8.78		
Within Populations	1322.457	2.13299	69.52		
Total	1656.579	3.06803			

Significance is based on 50,175 permutations.

### Effective population size and demographic changes

For the following analyses, localities were grouped into the following populations based on genetic cluster assignment: western population = Flabougou (site 1) and Niono (site 2), central population = Mopti (site 3) and Niamey (site 4), Lake Lere population = Lake Lere (site 5), and Lake Chad population = Lake Chad watershed (sites 6–16). Only one population, Lake Chad, showed evidence of a recent population bottleneck under the infinite allele model and the standardized differences test (*P* = 0.04234); however other mutation models and statistical tests did not produce significant results (Additional file 4: Table S3).

Current effective population size (*N*_*e*_) estimates varied between methods, but showed a general trend of the western population having the smallest *N*_*e*_ and Lake Chad having the largest *N*_*e*_, while the central and Lake Lere populations exhibited intermediate sizes (based on 95% CI) (Table 4). These differences in population size were also reflected in the genetic diversity measures, with the western population exhibiting the lowest level of genetic diversity, followed by Lake Lere, central, and Lake Chad (Table 4). Differences in sample size among populations could have influenced estimates of *N*_*e*_ and genetic diversity; however, the general trends across populations did not directly reflect sample size differences providing increased confidence in the results. Interestingly, despite having the smallest *N*_*e*_ and lowest genetic diversity estimates, the western population exhibited the highest proportion of private alleles (Table 4).

**Table 4:** **Summary of genetic diversity measures and** ***N***_***e***_ **for the four inferred populations of** ***Varanus niloticus***

**Population**	***H***_***o***_	***H***_***e***_	***N***_***a***_	***A***_***R***_	***PA***_***R***_	**Estimated Current** ***N***_***e***_		**Estimated Ancestral** ***N***_***e***_ **(95% CI)**
						**LD (95% CI)**	**MSVAR (95% CI)**	
Western	0.306	0.328	3.182	2.73	0.94	15.6, (7.3–42.5)	52.44, (0.02**–**52.45)	16,254.38, (1,375.04–16,987.52)
Central	0.375	0.340	3.182	2.81	0.20	∞, (24.2–∞)	143.90, (0.42**–**143.96)	11,168.20, (318.74–11,299.42)
Lake Lere	0.332	0.333	3.182	3.13	0.47	10.9, (2.3–∞)	218.33, (10.61**–**224.27)	46,212.13, (75.30**–**46,237.46)
Lake Chad	0.395	0.429	6.455	3.96	0.72	908.5, (336.0–∞)	1,373.27, (180.81**–**1,445.73)	2,781.28, (104.47–2,858.70)

*H*_*o*_ = observed heterozygosity; *H*_*e*_ = expected heterozygosity; *N*_*a*_ = number of different alleles; *A*_*R*_ = allelic richness; *PA*_*R*_ = private allelic richness. Estimated current *N*_*e*_ is based on the linkage disequilibrium (LD) method in NeEstimator as well as the coalescent-based method of MSVAR. The symbol ∞ indicates that no evidence exists for a genetic signature caused by a finite number of parents.

**Figure 5:**
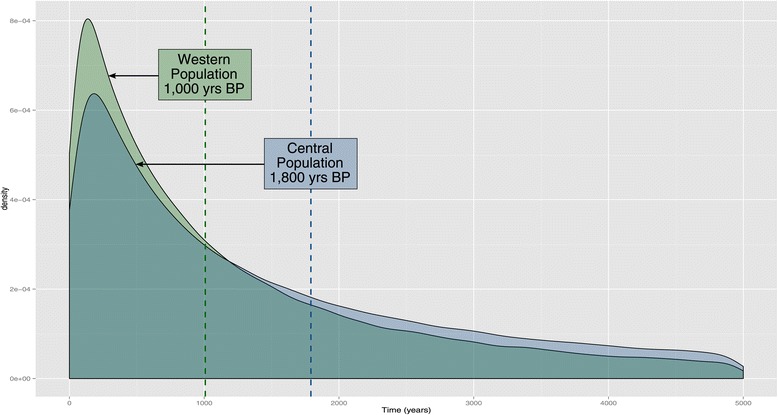
**Density plot of MSVAR results showing the timing of population declines.** The median values are indicated by the dotted vertical lines for the western (green) and central (blue) populations.

MSVAR results also indicated that *Varanus niloticus* populations have undergone demographic changes. Non-overlapping peaks in log(*N*_*e*_) for the western, central, and Lake Lere populations (Additional file 5: Figure S2), indicated historical population declines. The Lake Chad population, however, showed evidence of having a more stable population size. Ancestral *N*_*e*_ estimates are provided in Table 4, though our confidence in these values is lower due to their relatively flat density distributions (Additional file 5: Figure S2). The shifts in population size occurred approximately 1,000 (0.71–1,008.01) years BP for the western population and 1,800 (3.52–1,793.18) years BP for the central population (Figure 5) based on the median estimates. For Lake Lere, the density plot showing the approximate timing of demographic change does not reach a clear peak, indicating a different pattern of decline than the other populations, possibly a gradual decline that is presently continuing (Additional file 5: Figure S2).

### Gene flow and dispersal among populations

Our estimation of migration rates among *Varanus niloticus* populations indicated that the western and Lake Chad populations are fairly isolated, having a majority of their genetic contribution from within their own population (Table 5). Conversely, the central and Lake Lere populations, both located in the Niger watershed, exchanged a large number of migrants, having approximately one third of their genetic composition from the other population.

**Table 5:** **Bayesian assessment of migration rates among the four inferred** ***Varanus niloticus*** **populations**

	**Western**	**Central**	**Lake Lere**	**Lake Chad**
**Western**	0.990 (SD 0.010)			
**Central**		0.679 (SD 0.012)	0.303 (SD 0.018)	
**Lake Lere**		0.277 (SD 0.032)	0.690 (SD 0.022)	
**Lake Chad**				0.998 (SD 0.002)

Rates indicate gene flow from source populations (top) to sink populations (side) for the populations identified by clustering methods. Empty cells indicate migration rates < 0.05.

The isolation-by-distance analysis produced a significant result, showing that river distance explains a large degree of the genetic variation among *Varanus niloticus* localities (Table 6). However, the result showing localities exhibiting higher than expected *F*_*ST*_ values (Additional file 6: Figure S3) indicates that factors other than distance are likely contributing to the genetic structure within *V. niloticus*. To determine whether environmental heterogeneity is a factor in the observed genetic partitioning, annual precipitation, temperature seasonality, and elevation were examined while controlling for river distance (Table 6). These analyses generated non-significant results and therefore could not support the hypothesis that the environmental variables examined are contributing to the genetic structure within *V. niloticus*.

**Table 6:** **Isolation by river distance and environmental heterogeneity across** ***Varanus niloticus*** **collection localities**

**Variable**	***r***	***P*** **(** ***r*** **< = 0)**	***R*** ^***2***^
River distance (km)	0.7978	**< 0.0010**	0.637
Controlling for river distance:			
Annual Precipitation (−km)	−0.2559	0.9590	
Temperature Seasonality (−km)	0.1386	0.1210	
Elevation (−km)	−0.2598	0.9650	

The normalized Mantel’s statistic (*r*), significance [*P*(*r* < = 0)], and correlation coefficient (*R* ^*2*^) for the correlation between river distance (km) and genetic distance [*F*_*ST*_/ (1-*F*_*ST*_)] are provided. The bottom three rows show results from the Partial Mantel tests, controlling for river distance. The bold value corresponds to a significant correlation (P<0.05).

## Discussion

Although managed as a single taxonomic unit, the results of this study clearly show that *Varanus niloticus* exhibits a substantial degree of genetic partitioning throughout Sahelian Africa. Our results indicate considerably restricted gene flow between the westernmost population and the rest of the region, despite relatively short geographic distances (approximately 175 km between the western and central populations). The level of intraspecific differentiation found among *V. niloticus* populations throughout the Sahel is comparable to proposed distinct evolutionary lineages in other reptile species [42,43]. Mitochondrial sequence data from all populations matched voucher museum specimens of *V. niloticus* from respective localities (Dowell, unpublished dissertation), alleviating concerns of possible misidentification. Additionally, all sample localities are outside of the published distribution of the ornate monitor, *V. ornatus*, a morphologically similar species restricted to the forested regions of western and central Africa [44,45]. For comparison, sequences were also generated from *V. ornatus* museum material (Dowell, unpublished dissertation), further increasing our confidence in the sample identification. Future studies are necessary to determine whether other ecological, behavioral, or chromosomal factors are contributing to the reproductive isolation of the western population.

Contrary to our expectations, the largest genetic break occurs within a single drainage basin, near the Inner Niger Delta in Mali, an area characterized by floodplains and reticulating river networks. The Inner Niger Delta represents a suture zone for other Sahelian species, separating western and eastern lineages [46,47]. These congruent biogeographic patterns across species indicate that historic events likely shaped the evolutionary history of the Sahelian faunal community [48]. McIntosh [49] provides evidence that the Niger River stopped flowing during Pleistocene glacial periods and that the redistribution of sand caused large dunes to form in the region of the Inner Delta. When the Niger River began to flow again, these sand dunes acted as a dam, forming the large Paleo-lake Debo during the late Pleistocene and early Holocene [49,50]. The drying of the Niger River during glacial periods likely acted as a barrier to *Varanus niloticus* dispersal, and populations isolated in riverine refugia differentiated from one another.

Other evidence suggests that historic drainage patterns could have played a large role in shaping the present-day genetic patterns within *Varanus niloticus*. Individuals within the Senegal and Niger watersheds were assigned to the same genetic cluster. This low degree of genetic differentiation could be a reflection of the historic connection between the Senegal and Niger drainage basins, which occurred until the late Pleistocene [50], and likely facilitated past migration. Presently, the frequent flooding of these riverine systems has possibly allowed for the continuing exchange of migrants. On the other hand, the relatively low yet significant pairwise *F*_*ST*_ value between these localities suggests that gene flow might be slightly reduced, possibly due to the currently separate watersheds.

The genetic structure of the central, Lake Lere, and Lake Chad populations supports our hypothesis of reduced gene flow across present-day watershed boundaries. Our analysis of migration rates indicated a high degree of gene flow between the central and Lake Lere populations, located in the western and eastern drainages of the Niger basin, respectively. Although Lake Lere is in close proximity to locations within the Lake Chad watershed (shortest distance = 209 km), our analyses indicated reduced gene flow between these populations, evidenced by significant *F*_*ST*_ values and the spatial clustering analysis with BAPS. In contrast to BAPS, the STRUCTURE results showed a higher level of admixture and hierarchical partitioning among these populations; however, at higher *K* values (not shown) the distinctiveness of the Lake Lere population became more evident. The STRUCTURE analysis also showed additional sub-structuring among *Varanus niloticus* localities within the Lake Chad watershed. Sample sites along tributaries displayed differing proportions of ancestry than localities within Lake Chad itself. This could reflect source-sink dynamics of individuals dispersing downstream along tributaries and intermixing in Lake Chad, where the rivers converge.

Across methods, the western population showed evidence of having a small effective population size (*N*_*e*_) and lower genetic diversity compared to other *Varanus niloticus* populations in the region. This could be a result of the limited gene flow that we detected. Additionally, the prolonged intensive harvesting of *V. niloticus* in Mali [14] could be leading to reduced population sizes and lower genetic diversity in the region.

The decline in *Varanus niloticus* populations that occurred around 1,000–1,800 years BP corresponds to the drastic expansion and organization of human civilizations in West Africa [51]. Specifically, the ancient city of Jenné-jeno inhabiting the Inner Niger Delta region, expanded rapidly to cover an area of 330 km^2^ in 800–900 AD before eventually declining [51]. This ancient city was a major hub along the trans-Saharan trade route [51]. Archaeological excavations at the Jenné-jeno site recovered a high proportion of aquatic reptiles, including varanids, and this bias was mainly attributed to exploitation by fisherman [52]. Therefore, the negative effects of humans on *V. niloticus* populations in Sahelian Africa likely have a longer history than previously thought.

## Conclusion

Our findings have important implications for the management of Sahelian *Varanus niloticus* populations. Although listed on Appendix II of CITES, managing international trade of *V. niloticus* without population size and genetic structure information could be detrimental to the species. The four genetic groups that we identified in Sahelian Africa represent separate demographic units and possibly a distinct lineage based on private allelic richness and differing allele frequencies. These genetic groups provide an objective and useful delineation for managing populations of *V. niloticus* throughout Mali, Niger, and Chad. In the future, a genetic assessment of *V. niloticus* should be expanded to the full range of this species to enable more thorough monitoring of international trade and the ability to source harvested individuals from other locations.

This study highlights the utility of genetic methods for informing management practices and conservation decisions. Many CITES-listed species are currently managed without empirical data and could greatly benefit from species- and population-level assessments of genetic structure and diversity [53]. Monitor lizards, in particular, are all protected under CITES; however, information on population size and vulnerability status is lacking for a majority of varanid species [54]. Genetic tools can provide an effective means to detect genetic diversity loss, estimate effective population size, and identify independent population segments within harvested species [3,55].

While biodiversity data is limited throughout the Sahel, recent studies in this region have uncovered a high degree of endemism and species richness [56]. Similarly, genetic assessments have revealed cryptic diversity and genetic structuring in many Sahelian taxa [46,47,57-59]. With increasing aridity predicted for this region in future years [60] and continual destruction of habitat [8,56], genetic isolation and fragmentation of Sahelian species will likely increase. Therefore, understanding and protecting the complex patterns of biodiversity in the Sahel should be of great importance.

## Additional files

Additional file 1: Table S1. PCR protocols for each of the microsatellite loci used. Additional file 2: Table S2. Intraspecific genetic diversity estimates for each of the *Varanus niloticus* sample localities. Calculations include observed heterozygosity (*H*_*o*_), expected heterozygosity (*H*_*e*_), number of alleles per locus (*N*_*a*_), allelic richness (*A*_*R*_), and private allelic richness (*PA*_*R*_ *)* as well as loci showing deviations from Hardy-Weinberg equilibrium (HWE) with *P* <0.01. Locality numbers are consistent with Table 1. Additional file 3: Figure S1. Structure harvester results. Plots show the mean estimated Ln probability (A) and Δ*K* for *K* = 1–10 (B) and *K* = 2–10 (C). Additional file 4: Table S3. BOTTLENECK results for *Varanus niloticus* populations. Additional file 5: Figure S2. MSVAR results for each of the inferred populations of *Varanus niloticus*. Density plots show the log (*N*_*e*_), far left, and *N*_*e*_, central, of current (red) and ancestral (blue) population sizes. The density plots on the far right show the time since the change in *N*_*e*_ occurred. Additional file 6: Figure S3. Mantel test of genetic distance [*F*_*ST*_/ (1-*F*_*ST*_)] versus river distance.
