# Supplementary material for: Fine-scale genetic analysis of the exploited Nile monitor (Varanus niloticus) in Sahelian Africa
Source: BMC Genet. 2015 Mar 28;16:32. doi: 10.1186/s12863-015-0188-x (PMC4391116; doi:10.1186/s12863-015-0188-x)
Supplement: Additional file 1: Table S1. — PCR protocols for each of the microsatellite loci used. [file 12863_2015_188_MOESM1_ESM.docx]

**Additional file 1 -Table S1**

| **Locus** | **Annealing Temperature (°C)** | **PCR Conditions** | **Reference** |
| --- | --- | --- | --- |
| K7 | 50 | 94°C for 5 min  94°C for 40 sec  Annealing for 40 sec  65°C for 90 sec  65°C for 5 min | Ciofi & Bruford 1998  Ciofi et al. 2011 |
| K10 | 49 |  |  |
| K11 | 51 |  |  |
| K15 | 48 |  |  |
| K22 | 51 |  |  |
| K23 | 51 |  |  |
| VARSA07 | 49 | 94°C for 5 min  94°C for 30 sec  Annealing for 40 sec  72°C for 40 sec  72°C for 10 min |  |
| VARSA10 | 51 |  | Fu et al. 2011 |
| VA17  VA38  VA74 | 48  51  51 | 94°C for 5 min  94°C for 30 sec  Annealing for 40 sec  72°C for 40 sec  72°C for 10 min | Fitch et al. 2005 |
