# Supplementary material for: Fine-scale genetic analysis of the exploited Nile monitor (Varanus niloticus) in Sahelian Africa
Source: BMC Genet. 2015 Mar 28;16:32. doi: 10.1186/s12863-015-0188-x (PMC4391116; doi:10.1186/s12863-015-0188-x)
Supplement: Additional file 2: Table S2. — Intraspecific genetic diversity estimates for each of the Varanus niloticus sample localities. Calculations include observed heterozygosity (H o), expected heterozygosity (H e), number of alleles per locus (N a), allelic richness (A R), and private allelic richness (PA R ) as well as loci showing deviations from Hardy-Weinberg equilibrium (HWE) with P <0.01. Locality numbers are consistent with Table 1. [file 12863_2015_188_MOESM2_ESM.docx]

**Additional file 2 – Table S2**

| **Locality #** | ***H_o_*** | ***H_e_*** | ***N_a_*** | ***A_R_*** | ***PA_R_*** | **HWE** |
| --- | --- | --- | --- | --- | --- | --- |
| 1 | 0.350 | 0.320 | 2.636 | 1.96 | 0.28 |  |
| 2 | 0.273 | 0.295 | 2.545 | 1.85 | 0.21 |  |
| 3 | 0.371 | 0.341 | 3.182 | 2.01 | 0.04 |  |
| 4 | 0.394 | 0.268 | 1.727 | 1.73 | 0.00 |  |
| 5 | 0.332 | 0.333 | 3.182 | 2.04 | 0.17 |  |
| 6 | 0.428 | 0.428 | 3.636 | 2.35 | 0.10 | Varsa10 |
| 7 | 0.406 | 0.432 | 5.182 | 2.44 | 0.07 |  |
| 8 | 0.417 | 0.422 | 5.364 | 2.45 | 0.07 | K23 |
| 9 | 0.461 | 0.448 | 4.455 | 2.48 | 0.06 |  |
| 10 | 0.424 | 0.419 | 4.000 | 2.40 | 0.06 |  |
| 11 | 0.363 | 0.420 | 4.273 | 2.44 | 0.05 |  |
| 12 | 0.362 | 0.422 | 5.182 | 2.42 | 0.07 | K23 |
| 13 | 0.413 | 0.419 | 4.273 | 2.41 | 0.07 |  |
| 14 | 0.527 | 0.389 | 2.727 | 2.30 | 0.08 |  |
| 15 | 0.314 | 0.332 | 4.091 | 2.05 | 0.04 | K23 |
| 16 | 0.370 | 0.368 | 3.273 | 2.23 | 0.03 |  |
