# Supplementary figures and images for: Fine-scale genetic analysis of the exploited Nile monitor (Varanus niloticus) in Sahelian Africa
Source: BMC Genet. 2015 Mar 28;16:32. doi: 10.1186/s12863-015-0188-x (PMC4391116; doi:10.1186/s12863-015-0188-x)

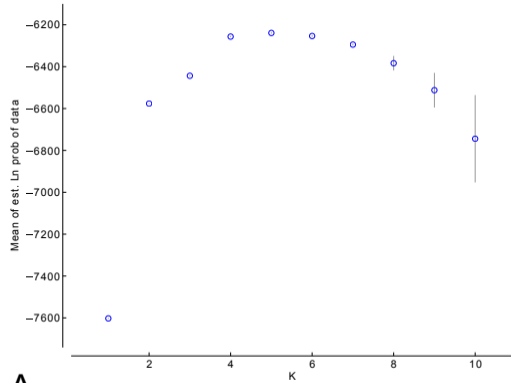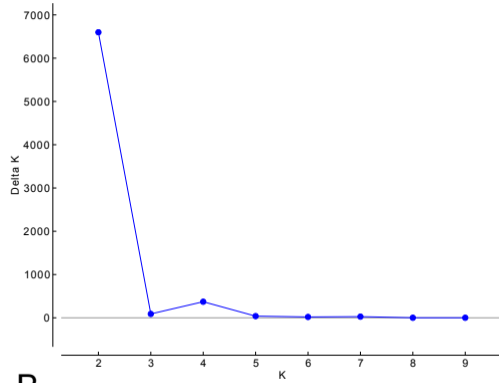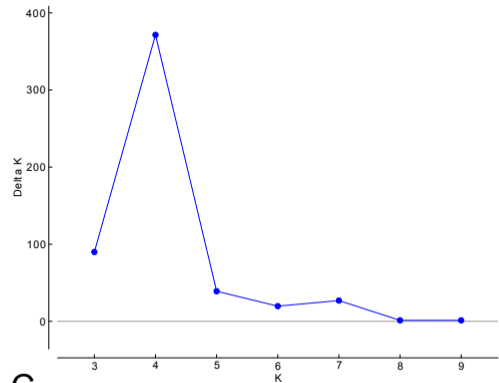

Supplement: Additional file 3: Figure S1. — Structure harvester results. Plots show the mean estimated Ln probability (A) and ΔK for K = 1–10 (B) and K = 2–10 (C). [file 12863_2015_188_MOESM3_ESM.pdf]

Western Population:

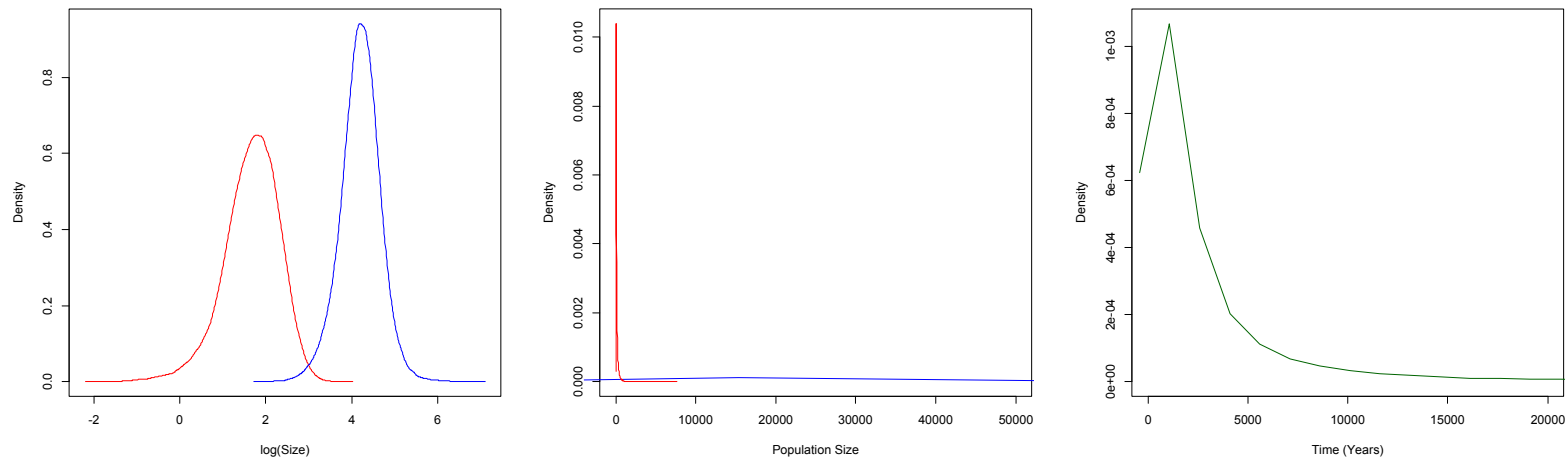

Central Population:

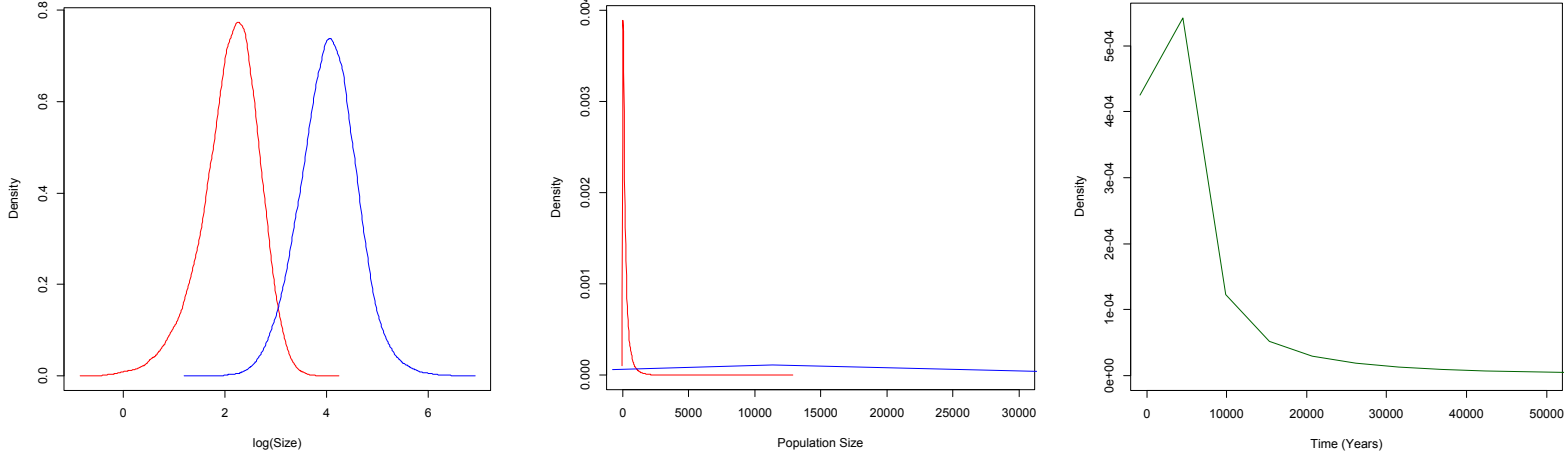

Lake Lere Population:

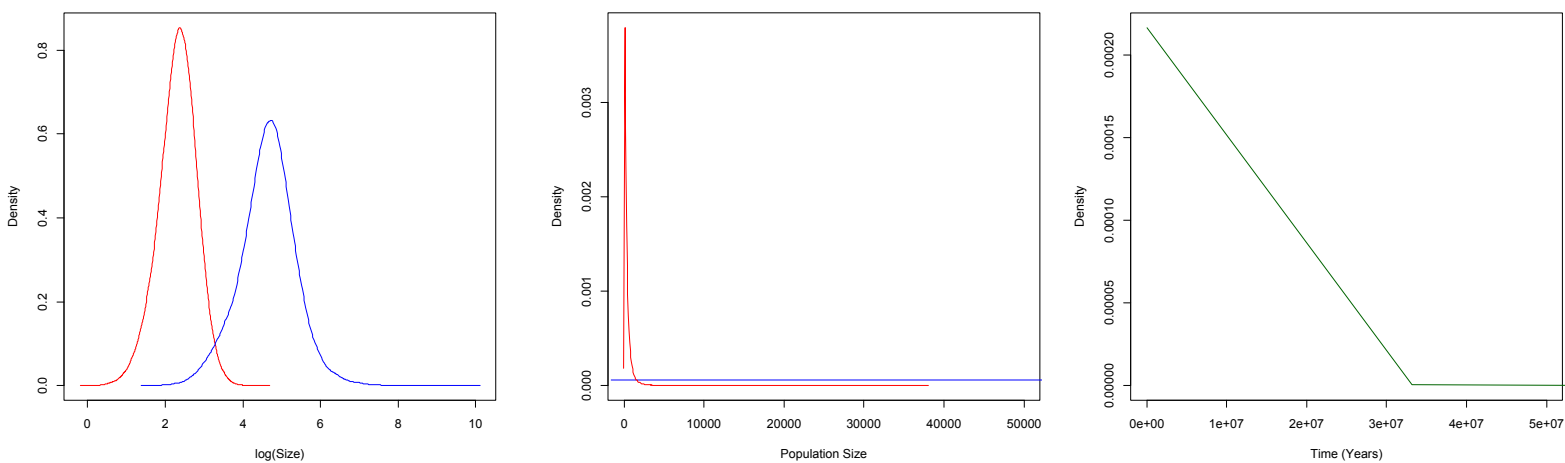

Lake Chad Population:

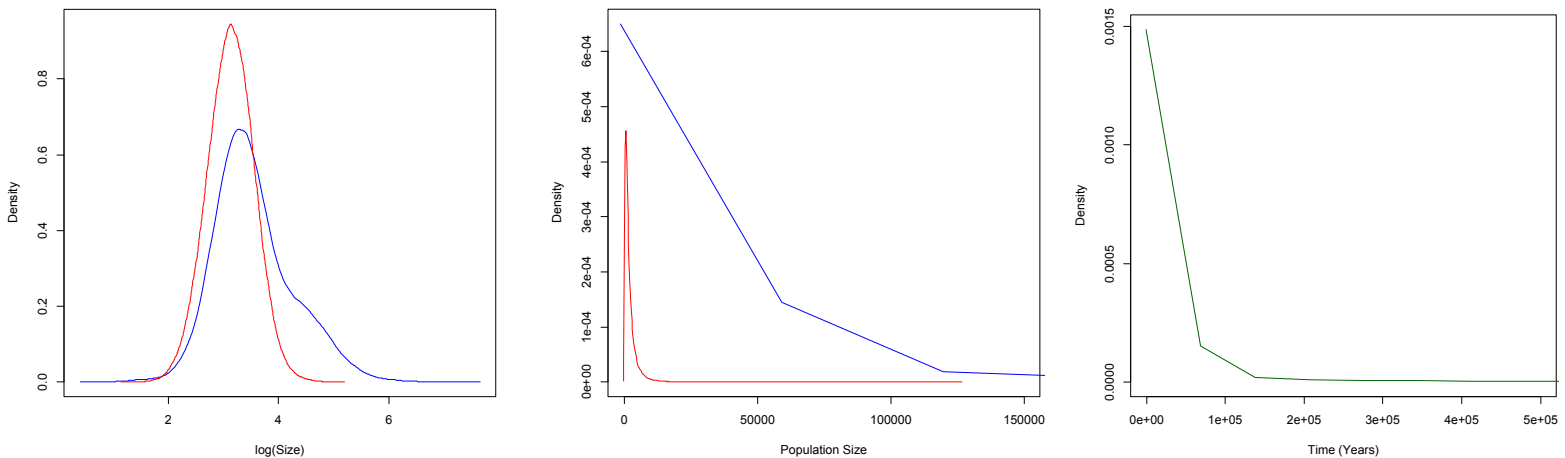

Supplement: Additional file 5: Figure S2. — MSVAR results for each of the inferred populations of Varanus niloticus. Density plots show the log (N e), far left, and N e, central, of current (red) and ancestral (blue) population sizes. The density plots on the far right show the time since the change in N e occurred. [file 12863_2015_188_MOESM5_ESM.pdf]

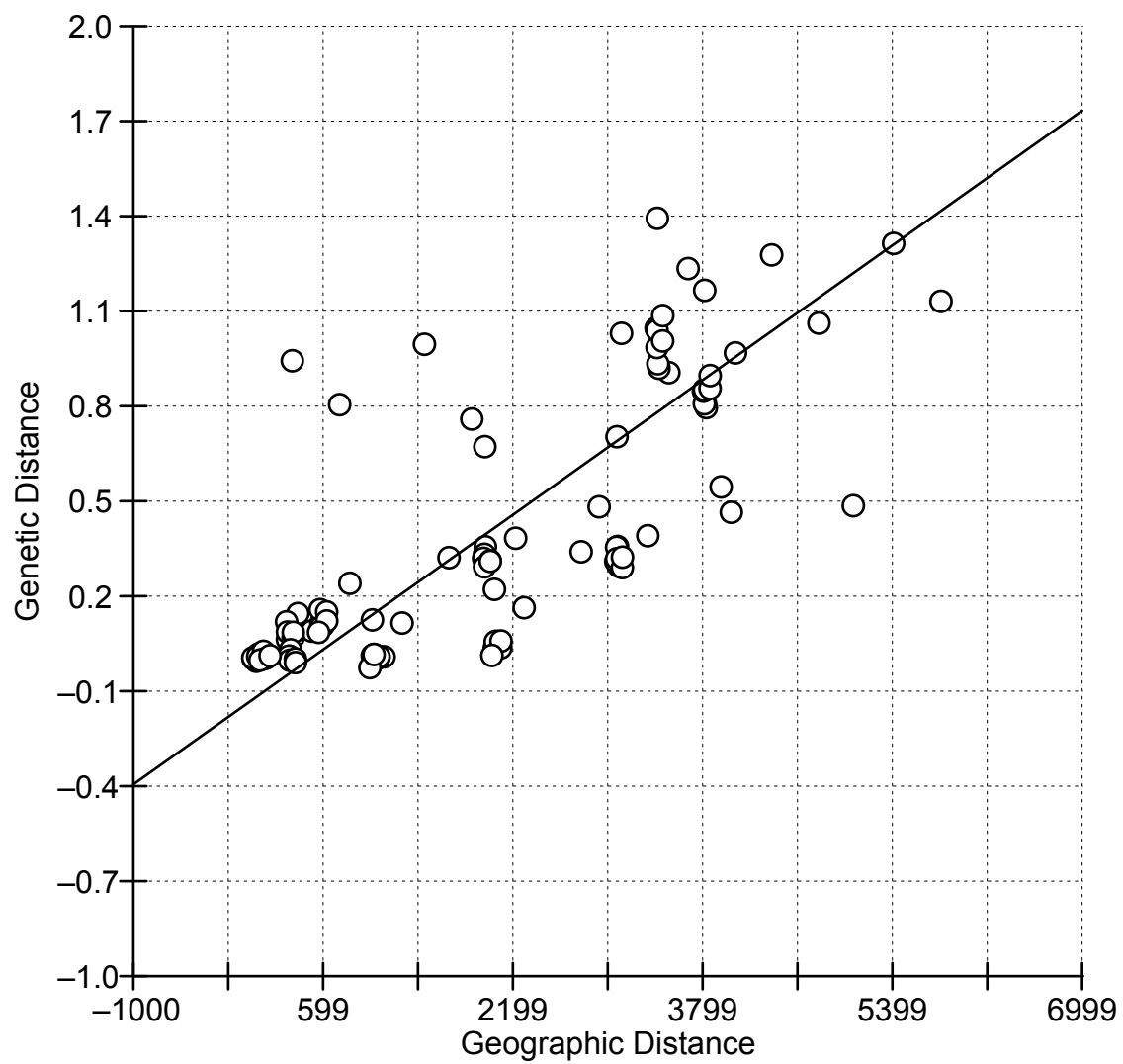

Supplement: Additional file 6: Figure S3. — Mantel test of genetic distance [F ST/ (1-F ST)] versus river distance. [file 12863_2015_188_MOESM6_ESM.pdf]
