# Supplementary material for: Fine-scale genetic analysis of the exploited Nile monitor (Varanus niloticus) in Sahelian Africa
Source: BMC Genet. 2015 Mar 28;16:32. doi: 10.1186/s12863-015-0188-x (PMC4391116; doi:10.1186/s12863-015-0188-x)
Supplement: Additional file 4: Table S3. — BOTTLENECK results for Varanus niloticus populations. [file 12863_2015_188_MOESM4_ESM.docx]

**Additional file 4 – Table S3**

| **Population** | **Mutation Model** | **Sign Test**  **(*P*-value)** | **Standardized Differences Test** | **Wilcoxon Sign Rank Test**  **(*P*-values)** |
| --- | --- | --- | --- | --- |
| Western | IAM | 0.29267 | T2: 1.209  *P*-value: 0.11343 | Deficiency: 0.85156  Excess: 0.18750  Two-tail: 0.37500 |
|  | SMM | 0.33724 | T2: -1.407  *P*-value: 0.07970* | Deficiency: 0.23438  Excess: 0.81250  Two-tail: 0.46875 |
|  | TPM (70%SMM) | 0.62694 | T2: 0.145  *P*-value: 0.44225 | Deficiency: 0.65625  Excess: 0.40625  Two-tail: 0.81250 |
| Central | IAM | 0.53952 | T2: 0.380  *P*-value: 0.35191 | Deficiency: 0.71484  Excess: 0.32617  Two-tail: 0.65234 |
|  | SMM | 0.05293* | T2: -2.054  *P*-value: 0.01997* | Deficiency: 0.10156  Excess: 0.91797  Two-tail: 0.20313 |
|  | TPM (70%SMM) | 0.41667 | T2: -0.678  *P*-value: 0.24884 | Deficiency: 0.32617  Excess: 0.71484  Two-tail: 0.65234 |
| Lake Lere | IAM | 0.28880 | T2: 0.536  *P*-value: 0.29590 | Deficiency: 0.76563  Excess: 0.28906  Two-tail: 0.57813 |
|  | SMM | 0.40581 | T2: -1.438  *P*-value: 0.07526* | Deficiency: 0.18750  Excess: 0.85156  Two-tail: 0.37500 |
|  | TPM (70%SMM) | 0.38422 | T2: -0.392  *P*-value: 0.34736 | Deficiency: 0.40625  Excess: 0.65625  Two-tail: 0.81250 |
| Lake Chad | IAM | 0.34493 | T2: 1.724  *P*-value: **0.04234** | Deficiency: 0.93457  Excess: 0.08008  Two-tail: 0.16016 |
|  | SMM | 0.10792 | T2: -2.780  *P*-value: 0.00272* | Deficiency: 0.06543*  Excess: 0.94727  Two-tail: 0.13086 |
|  | TPM (70%SMM) | 0.57662 | T2: 0.055  *P*-value: 0.47824 | Deficiency: 0.46094  Excess: 0.57715  Two-tail: 0.92188 |
